# Inhibition of STAT3/VEGF/CDK2 axis signaling is critically involved in the antiangiogenic and apoptotic effects of arsenic herbal mixture PROS in non-small lung cancer cells

**DOI:** 10.18632/oncotarget.21973

**Published:** 2017-10-19

**Authors:** Hyemin Lee, Hyo-Jung Lee, Ill Ju Bae, Jeong Jin Kim, Sung-Hoon Kim

**Affiliations:** ^1^ Cancer Molecular Targeted Herbal Research Center, College of Korean Medicine, Kyung Hee University, Seoul, 130-701, Republic of Korea; ^2^ Chunjisan Co. Ltd., 2804 Bangbae-dong, Seocho-gu Seoul 137-853, Republic of Korea

**Keywords:** arsenic herbal mixture, non-small cell lung cancer, apoptosis, angiogenesis, STAT3

## Abstract

Despite the antitumor effects of asrsenic trioxide (As2O3), tetraarsenic hexoxide (As4O6 or PR) and tetraarsenic tetrasulfide (As4S4) in several cancers, their adverse poisoning, toxicity and resistance are still hot issues for effective cancer therapy. Here, antitumor mechanism of arsenic herbal mixture PROS including PR and OS (Oldenlandia diffusa and Salvia miltiorrhiza extract) was elucidated in non-small cell lung cancer cells (NSCLCs), since PR alone showed resistant cytotoxicity in NSCLCs compared to other cancers. PROS exerted significant cytotoxicity, induced sub-G1 phase and S phase arrest, increased apoptotic bodies, and attenuated the expression of pro-PARP, Bcl-2, Cyclin E, Cyclin A, CDK2, E2F1, p-Src, p-STAT3, p-ERK, p-AKT, COX-2 and SOCS-1 in A549 and H460 cells along with disrupted binding of STAT3 with CDK2 or VEGF. Notably, PROS inhibited VEGF induced proliferation, migration and tube formation in HUVECs and suppressed angiogenesis in chorioallantoic membrane (CAM) assay via reduced phosphorylation of VEGFR2, Src and STAT3. Consistently, PROS reduced the growth of H460 cells implanted in BALB/c athymic nude mice via inhibition of STAT3, and VEGF and activation of caspase 3. Overall, these findings suggest that PROS exerts antiangiogenic and apoptotic effects via inhibition of STAT3/ VEGF/ CDK2 axis signaling as a potent anticancer agent for lung cancer treatment.

## INTRODUCTION

Lung cancer is the most common cause of cancer related deaths and its main primary types are small lung cancer (10~15%) and non-small lung cancer (85~90%) worldwide [1, 2]. In general, the treatment for lung cancer is surgery, chemotherapy, radiotherapy, immunotherapy and targeted therapy mainly for EGFR, VEGF, ALK and NF-κB [3, 4].

It is well documented that arsenic trioxide (As_2_O_3_) exerts antitumor effect in several cancers including leukemia [5], breast cancer [6], prostate cancer [7], lung cancer [8], cervical cancer [9], gastric cancer [10, 11], pancreatic cancer [12], hepatocellular cancer [13] and glioblastoma [14]. Thus, Trisenox as a trade name of arsenic trioxide from Cephalon Biopharmaceutical Company has been applied to treat acute promyelocytic leukemia (APL) as a chemotherapeutic agent approved by the US FDA [15]. Recently tetraarsenic hexoxide(As_4_O_6_) showed antitumor effect more effectively than arsenic trioxide in HPV 16-positive SiHa cervical cancer [16] and enhanced radiation sensitivity to reduce the growth of Meth-A induced fibrosarcoma and nasopharyngeal squamous cancer implanted in Balb/c nu/nu mice [17]. Nevertheless, new combination therapy has been attractive with low dose of arsenic trioxide and natural compounds such as naringenin [18], resveratrol [19], retinoic acid [20] and lipoic acid [21], since the toxicity and poisoning of arsenic trioxide were reported as cardiac toxicity [22, 23], reproductive toxicity [24], hepatotoxicity [25] and resistance [26, 27]. As known as anticancer herbal medicines, *Oldenlandia diffusa* is known to have anti-cancer [28], antioxidant [29], anti-inflammatory [30] and immunomodulatory [31] and also *Salvia miltiorrhiza* has anti-inflammatory [32], antioxidant [33] and anti-cancer activity [34] with constituents of diterpene quinone, tanshinoneI, tanshinone II cryptotanshinone, and miltirone [35].

Thus, in the same line, the aim of the present study is to elucidate antitumor mechanism of PROS, an arsenic herbal mixture of tetraarsenic hexoxide (PR) and ethanol extract of *Oldenlandia diffusa* and *Salvia miltiorrhiza* (OS) in association with angiogenesis and apoptosis in non-small cell lung cancer cells (NSCLCs), since PR showed resistant cytotoxicity in A549 and H460 NSCLCs compared to other cancers such as HCT-116 colon cancer, 786-O renal cancer, PC-3 and DU145 prostate cancers.

## RESULTS

### PROS exerted significant cytotoxicity in A549 and H460 NSCLCs better than PR or OS alone

**Figure 1:**
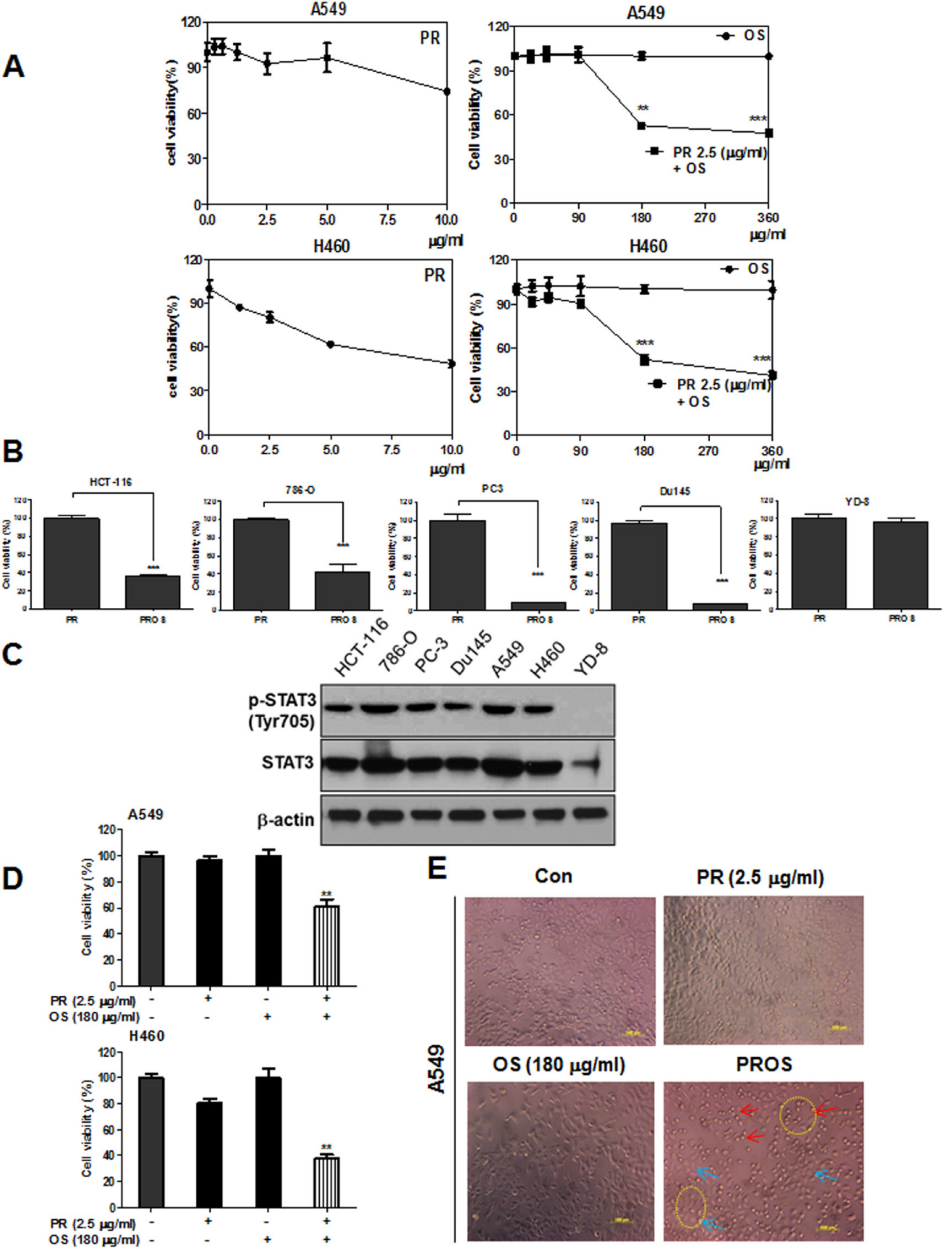
PROS exerted significant cytotoxicity in A549 and H460 NSCLCs better than PR or OS (**A**) Cytotoxic effect of PROS (PR (2.5 μg/ml and OS (180 μg/ml) in A549 and H460 NSCLCs better than PR or OS. A549 and H460 cells were seeded onto 96-well microplates at a density of 1 × 10^4^ cells/well and treated with PR, OS and PROS for 24 h. Then cell viability was determined by MTT assay. (**B**) Cytotoxic effect of PROS in several cancer cell lines (HCT-116: human colon cancer, 786-O: human renal cancer, PC-3 and Du145: human prostate cancer, YD-8: human oral squamous cells). These cancer cells were seeded onto 96-well microplates at a density of 1 × 10^4^ cells/well and treated with PROS for 24 h. Then cell viability was determined by MTT assay. (**C**) The expression levels of p-STAT3 (Tyr705) and total-STAT3 in various cancer cell lines (HCT-116, 786-O, PC3, Du145, A549, H460 and YD-8) by Western blotting using anti-STAT3 (Tyr705) and total-STAT3 antibodies. (**D**) Synergistic effect of PROS on the viability and morphological changes in A549 and H460 cells. A549 and H460 cells were treated with PROS for 24 h and cell viability was determined by MTT assay. (**E**) Also, the morphology of PROS treated A549 cells was observed under microscope. Photographs were taken at Magnification ×200. Arrows indicate the live cells (blue) and dead cells (red). Data represent means ± SD. ^**^ *P* < 0.01 and ^***^P < 0.001 versus PR treated control.

Though PR or tetraarsenic hexoxide (As_4_O_6_) showed cytotoxic effect in several cancers such as SW620 colon cancer [36, 37], CaSki cervical cancer [16], U87MG malignant glioma cells [38], U937 leukemia [39] with IC_50_ of ~ 0.4 μg/ml (1 μM) , PR exhibited weak cytotoxicity in A549 and H460 NSCLCs with IC_50_ of over 4 μg/ml (10 μM), respectively, while OS did not show any toxicity up to 360 μg/ml (Figure 1A). However, OS initiated synergistic cytotoxicity with 2.5 μg/ml (6.3 μM) PR in A549 and H460 NSCLCs from 180 μg /ml (Figure 1A). In contrast, PROS, combination of 2.5μg/ml PR and 180 μg/ml OS, exerted significant cytotoxicity in A549 and H460 NSCLCs, HCT-116 colon cancer, 786-O renal cancer, PC-3 and DU145 prostate cancers compared to PR alone (Figure 1B and 1D), but not in YD-8 oral squamous cells (Figure 1B). To test expression of p-STAT3 (Tyr705) and total-STAT3 in HCT-116, 786-O, PC3, Du145, A549, H460 and YD-8 cells, Western blotting was performed using an anti-STAT3 (Tyr705) and total-STAT3 antibodies. As shown in Figure 1C, p-STAT3 was highly expressed in HCT-116, 786-O, PC3, Du145, A549 and H460, while that was low expressed in YD-8 cell lines (Figure 1C). Likewise, apoptotic morphology was shown only in PROS treated cells compared to OS or PR alone treated control cells (Figure 1E).

### PROS induced sub G1 accumulation, S phase arrest, attenuated the expression of pro-PARP, Bcl-2, p-ERK, cyclin E, cyclin A, Cdk-2 and E2F1 in A549 or/and H460 cells

**Figure 2:**
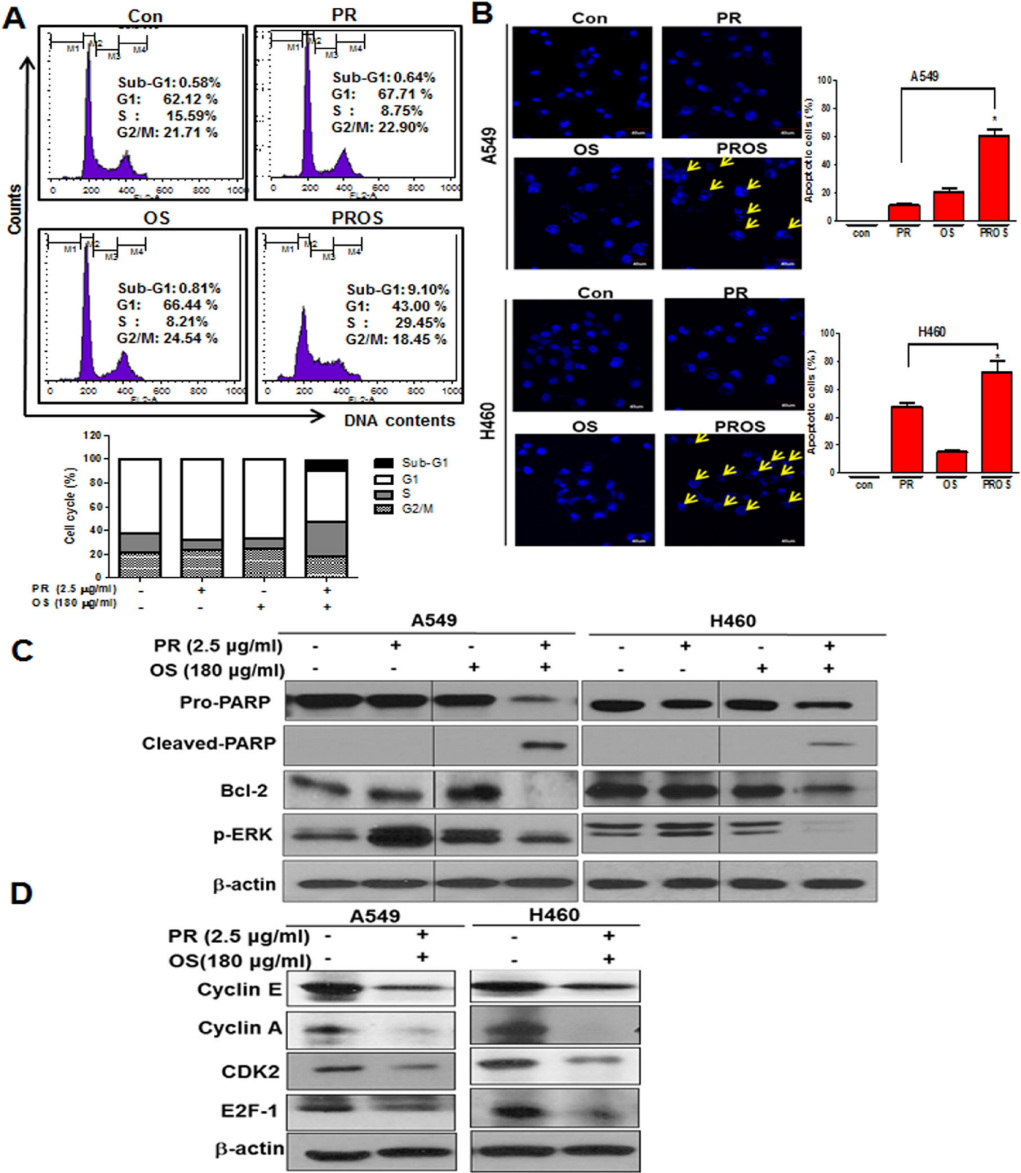
PROS induced apoptosis and S phase arrest in A549 or/and H460 NSCLCs (**A**) Effect of PROS on cell cycle distributions in A549 cells. Cell cycle analysis was performed in PR, OS and PROS treated A549 cells for 24 h by flow cytometry. Bar graphs represent the percentages of cell cycles DNA contents. (**B**) A549 and H460cells were seeded at a density of 4 × 10^5^ cells/ml and treated with PR, OS and PROS for 24 h. DAPI staining was conducted and visualized under fluorescence microscopy (×200). Yellow arrows indicate nuclear fragments. Bar graphs represent the percentages of apoptotic cells. Data represent means ± SD. ^*^P < 0.05 versus PR treated control. (**C**) Effect of PROS on apoptosis related proteins in A549 and H460 cells. Cells were treated with PROS for 24 h. Cell lysates were prepared and subjected to Western blotting for PARP, cleaved-PARP, Bcl-2 and p-ERK. (**D**) Effect of PROS on the expression of S phase arrest-related proteins in A549 and H460 cells. Cells were treated with PROS for 24 h. Cell lysates were prepared and subjected to Western blotting with antibodies against cyclin E, cyclin A, CDK2, and E2F-1.

To confirm the apoptotic effect of PROS, cell cycle analysis and DAPI staining were conducted in A549 cells treated by PROS compared to PR or OS alone. PROS increased sub-G1 population, induced S phase arrest to 29.45% compared to PR (8.75%) or OS (8.21%) (Figure 2A) and increased apoptotic bodies compared to PR or OS alone in A549 and H460 cells (Figure 2B). Likewise, PROS attenuated the expression of pro-PARP, Bcl-2, p-ERK (Figure 2C), cleaved PARP and also suppressed the expression of Cyclin E, Cyclin A, CDK2 and E2F1 as cell cycle related genes in A549 and H460 cells (Figure 2D).

### PROS suppressed the expression of p-STAT3, p-ERK, p-Src, p-AKT, COX-2, SOCS-1 and disrupted the binding of STAT3 with CDK2 or VEGF in NSCLCs

**Figure 3:**
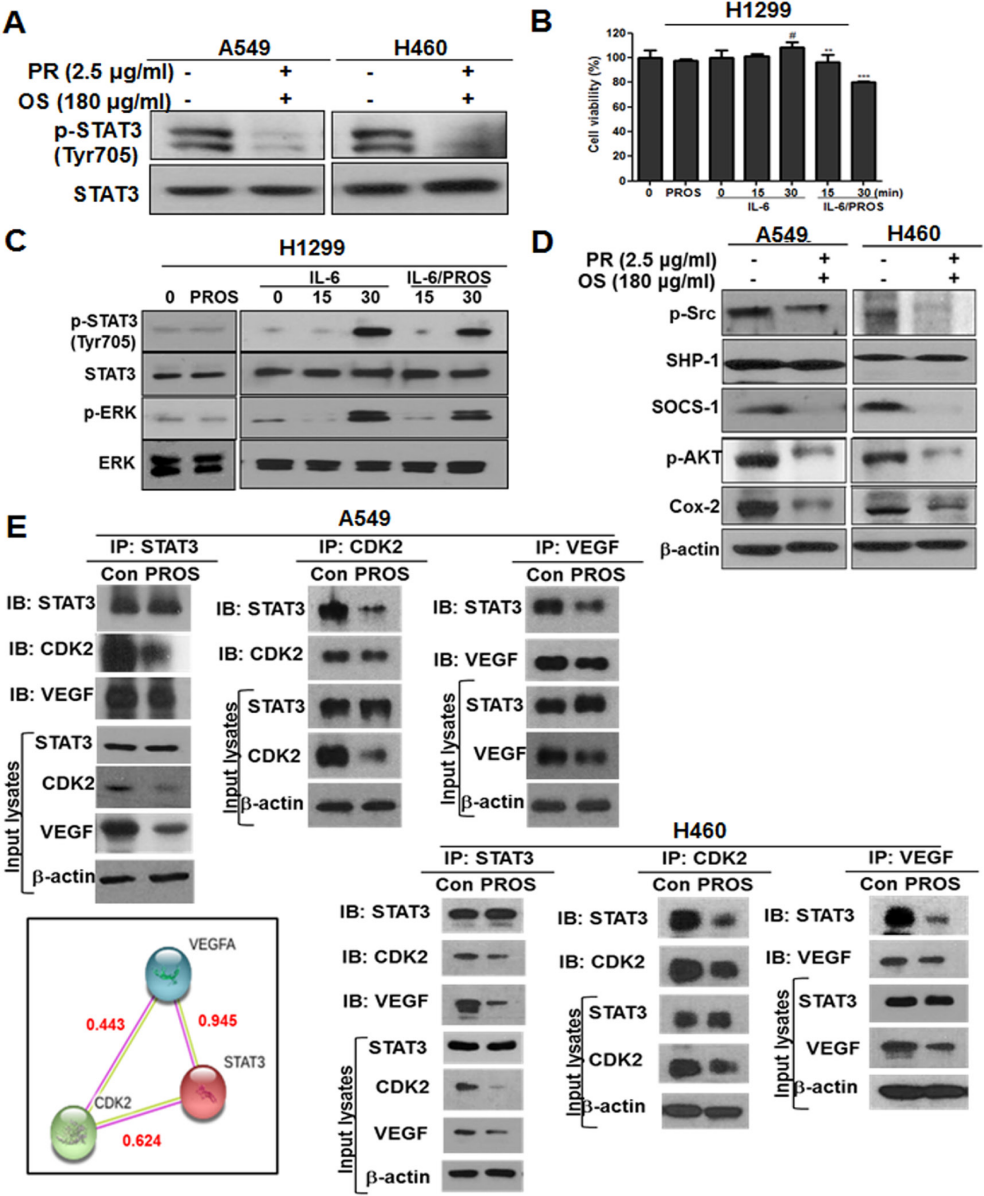
PROS inhibited phosphorylation of STAT3 and subsequently suppressed its interaction with CDK2 and VEGF in A549 and H460 or H1299 NSCLCs (**A**) Effect of PROS on p-STAT3 in A549 and H460 cells. Cells were treated with PROS for 24 h and cell lysates were subjected to Western blotting for p-STAT3 and STAT3. (**B**) H1299 cells were seeded onto 96-well microplates at a density of 1 × 10^4^ cells/well and treated with PROS for 24 h with or without IL-6 (20 ng/ml) stimulation. Then, MTT assay was performed. Data represent means ± SD. ^#^*p* < 0.05 vs untreated control, ^**^P < 0.01, ^***^P < 0.001 vs IL6 treated control. (**C**) Effect of PROS on p-STAT3 in IL6 treated H1299 cells. H1299 cells were treated with PROS for 24 h and then stimulated with IL-6 (20 ng/ml) for the indicated times. Cell lysates were subjected to Western blotting with antibodies against p-STAT3, STAT3, p-ERK, and ERK. (**D**) Effect of PROS on STAT3 related proteins in A549 and H460 cells. Cell lysates were subjected to Western blotting with antibodies against p-Src, SHP-1, SOCS-1, p-AKT, and COX-2. (**E**) Effect of PROS on STAT3 interacting proteins in A549 and H460 cells. A549 and H460 cells were treated with PROS for 24 h. STAT3/CDK2/VEGF interactions were analyzed in A549 and H460 cells by immunoprecipitation using antibodies against STAT3, CDK2, VEGF, followed by immunoblots with the indicated antibodies. Western-blot analysis was performed to detect STAT3, CDK2 and VEGF in input lysates. Input lysates indicate 5% pre-immunoprecipitated samples and b-actin levels confirm equivalent protein loading. Protein-Protein interaction networks by STRING database. STAT3 interacts with CDK2 and VEGF by STRING database. Red text (interaction score). Different line colors represent the types of evidence for the association.

PROS reduced phosphorylation of signal transducer and activator of transcription 3 (STAT3) (Tyr705) as a transcription factor in A549 and H460 cells (Figure 3A) and also attenuated the phosphorylation of interleukin 6 (IL-6) activated ERK and STAT3 in STAT3 low level H1299 cells (Figure 3C). Likewise, PROS reduced the cell viability of IL-6-stimulated H1299 cells (Figure 3B). Consistently, PROS reduced the expression of p-Src, p-AKT, cyclooxygenase 2 (COX-2) as survival genes and suppressor of cytokine signaling 1 (SOCS-1) that promotes tumor progression and toll like receptor (TLR) signaling [40] , but not SHP-1, in A549 and H460 cells (Figure 3D). Additionally, IP revealed that PROS attenuated the expression of VEGF and CDK2 and also disrupted the binding of STAT3 with CDK2 or VEGF after the treatment with PROS for 24 h in A549 and H460 cells (Figure 3E), while the score of protein-protein interaction (PPI) between STAT3 and CDK2, STAT3 and VEGF or VEGF and CDK2 were 0.624, 0.945 and 0.443, respectively.

### PROS inhibited angiogenesis via inhibition of p-VEGFR2/p-SRC/p-STAT3 axis signaling

**Figure 4:**
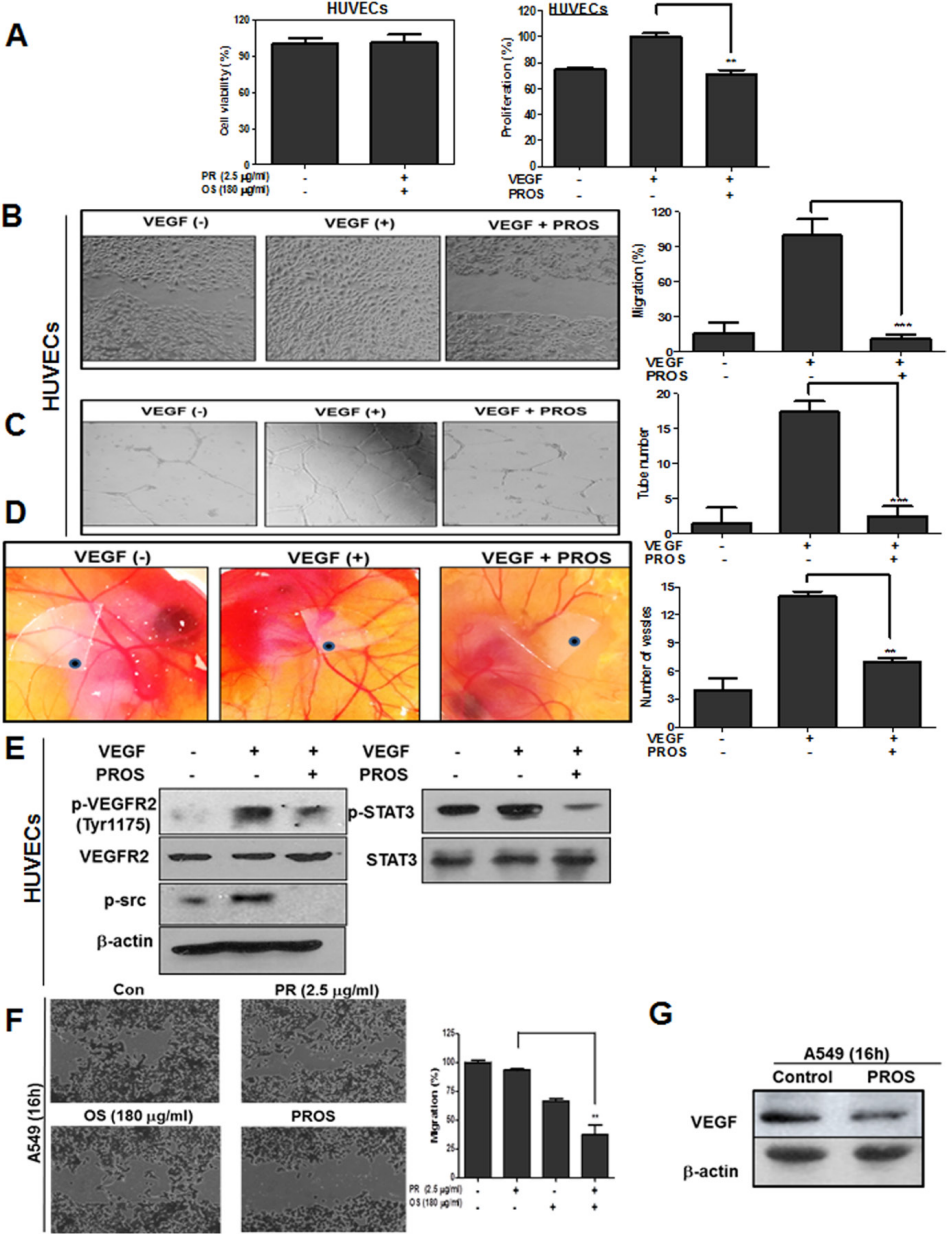
PROS suppressed VEGF-induced angiogenesis in HUVECs (**A**) Cytotoxic effect of PR, OS and PROS in VEGF treated HUVECs. Cytotoxic effect of PROS in HUVEC cells. Cells were plated onto 96-well microplate at a density of 1 × 10^4^ cells/well and treated with PROS for 24 h. Then the cell viability was assessed by MTT assay. Serum-starved HUVECs were plated onto 96-well microplate at a density of 1 × 10^3^ cells/well and exposed to PROS in M199 containing 5% heat-inactivated FBS, heparin (5 unit/ml) and VEGF 10 ng/ml for 48 h, and the proliferation was assessed by MTT assay. Data represent means ± SD. ^**^P < 0.01 versus VEGF-treated control. (**B**) Effect of PROS on VEGF-induced migration in HUVECs. HUVEC cells were scratched and then treated with PROS and/or VEGF (10 ng/ml) for 18 h. The number of cells migrated into the scratched area was photographed (×100) and calculated as a percentage of untreated control. Data represent means ± SD. ^***^P < 0.001 versus VEGF-treated control. (**C**) Effect of PROS on VEGF-induced tube formation in HUVECs. HUVECs (2 × 10^4^ cells) were seeded onto Matrigel-coated 24-well plates, and were cultured in M199 with 1% FBS, 10 ng/ml VEGF and 5 units/ml heparin with or without PROS for 18 h. Then, cells were fixed with 4% paraformaldehyde, and randomly chosen fields were photographed under an Axiovert S 100 light microscope (Carl Zeiss, Thornwood, NY, U.S.A.) at 100× magnification. Data represent means ± SD. ^***^P < 0.001 versus VEGF-treated control. (**D**) Effect of PROS on VEGF-induced angiogenesis in CAM. VEGF (100 ng/egg) entrapped in type I collagen gels were loaded on the chick chorioallantoic membranes (CAMs) of day 10 chick embryos. After 48 h incubation, a fat emulsion was injected under the CAMs for better visualization of the vessels. Neovascularization around disk in CAM was photographed. Data represent means ± SD. ^**^P < 0.01 versus VEGF-treated control. (**E**) Effect of PROS on angiogenesis related proteins in VEGF treated HUVECs. HUVEC cells were treated with PROS and/or VEGF (10 ng/ml) for 24 h. Cell lysates were subjected to Western blotting for p-VEGFR2, VEGFR2, p-STAT3, p-AKT and cyclinD1. (**F**) Effect of PROS on migratory activity in A549 cells. A549 cells were scratched and then treated with PROS for 16 h. The number of migrated cells into the scratched area was photographed (×100) and calculated as a percentage of untreated control. Data represent means ± SD. ^**^P < 0.01 vs PR treated control. (**G**) Effect of PROS on VEGF expression in A549 cells. A549 cells were treated with PROS for 16 h. Cell lysates were subjected to Western blotting for VEGF.

PROS inhibited VEGF induced proliferation, migration and tube formation in HUVECs at nontoxic concentration (Figure 4A–4C). Consistently, PROS suppressed neovascularization in CAM assay (Figure 4D). Furthermore, PROS reduced phosphorylation of VEGFR2, Src and STAT3 in VEGF treated HUVECs (Figure 4E). Consistently, PROS suppressed the migratory activity in A549 cells compared to PR or OS alone along with reduced expression of VEGF (Figure 4F and 4G).

### PROS abrogated the growth of H460 cells implanted in BALB/c athymic nude mice via inhibition of STAT3 and VEGF and activation of caspase 3

**Figure 5:**
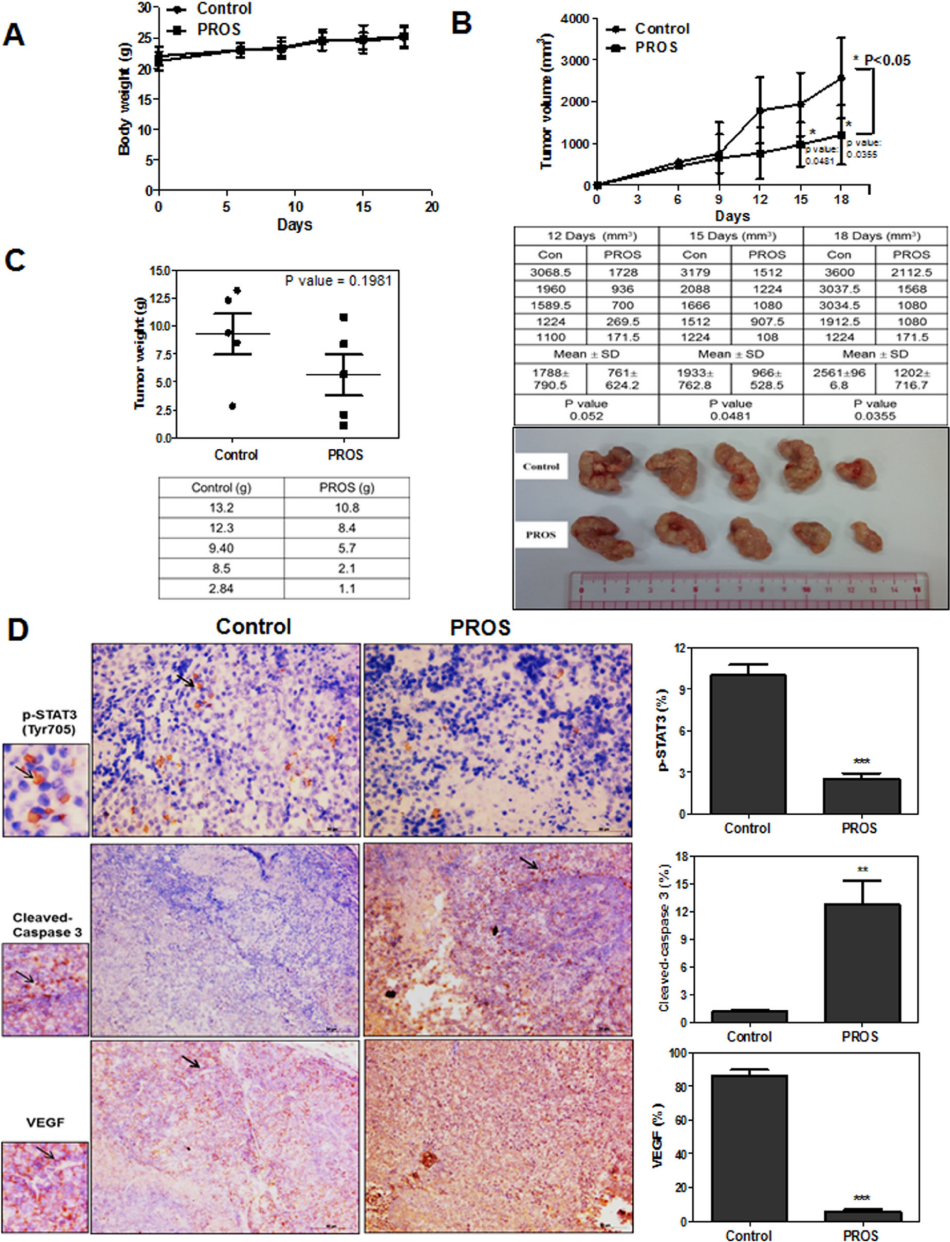
PROS reduced the growth of H460 cells implanted in BALB/c athymic nude mice (**A**) Body weights and (**B**) tumor volumes were monitored, while PROS was given daily by oral gavage for 18 days. Table represents the raw data of tumor volumes. ^*^p < 0.05 vs vehicle treated control. (**C**) Final tumor weights and photographs on Day 18 after tumor inoculation. Table represents the raw data of tumor weights. (**D**) Immunohistochemistry for p-STAT3, cleaved caspase3 and VEGF in tumor sections. Arrows indicate the immunostained cells for each antibody. Graphs show the levels of p-STAT3, cleaved caspase3 and VEGF in tumor sections. All stained sections were photographed under an Axiovert S 100 light microscope (Carl Zeiss, Inc., USA) at 200× magnifications. ^**^p < 0.01 and ^***^ *P* < 0.001 vs vehicle treated control.

To confirm *in vitro* antiangiogenic and apoptotic effect of PROS, animal study was conducted in H460 xenograft model. PROS did not affect body weight of mice (Figure 5A) and significantly reduced the tumor volumes (Figure 5B) and weights (Figure 5C) compared to untreated control. Furthermore, immunohistochemistry revealed that the expression of p-STAT3 and VEGF was suppressed and cleaved caspase 3 was activated in tumor sections isolated from PROS treated group mice compared to untreated control (Figure 5D).

## DISCUSSION

Emerging evidences revealed that arsenic compounds such as asrsenic trioxide (As_2_O_3_) [6, 15], tetraarsenic hexoxide (As_4_O_6_ or PR) [38, 39] and tetraarsenic tetrasulfide (As_4_S_4_) [41, 42] have antitumor effects in several cancers including acute promyelocytic leukemia (APL). Currently Trisenox (asrsenic trioxide injection) from Cephalon Pharmaceutical Company has been commercially used for APL treatment [43, 44], though it shows complication indications such as APL differentiation syndrome [45], cardiac conduction abnormalities [46] and hepatotoxicity [47]. Recently combination therapy of arsenic trioxide and natural compounds such as cucurbitacine B [48], cryptotanshinone [49], naringenin [18], resveratrol [19], retinoic acid [20] and lipoic acid [21] is recommended for synergistic antitumor effect for reducing adverse effect of arsenic compounds [50]. Here the antitumor mechanism of PROS, an arsenic herbal mixture that has been used for several cancers at KMD clinics, was explored in NSCLCs. PROS exerted significant cytotoxicity in several cancers such as A549, H460, HCT-116, 786-O, PC-3 and DU145 cancers compared to OS or PR alone, while PR showed weaker cytotoxicity in A549 and H460 NSCLCs compared to other cancers. Also, PROS induced sub G1 accumulation, S phase arrest, increased apoptotic bodies, and attenuated the expression of pro-PARP in A549 and H460 cells, indicating the apoptotic effect and S phase arrest of PROS. Consistently, PROS suppressed the expression of cell cycle related proteins such as Cyclin E, Cyclin A, CDK2, E2F1 in A549 and H460 cells, since *Cyclin E* binds to CDK2, which is required for the transition from G1 to S phase, Cyclin A can regulate cell cycle through S and G2/M phases with CDK2 or CDK1 [51] and also E2F1 is working during the G1/S transition. Also, as antiapoptotic proteins, the expression of Bcl-2, p-Src, p-ERK, p-AKT, COX-2 and SOCS-1 was attenuated in PROS treated A549 and H460 cells, implying PROS inhibits antiapoptotic proteins, leading to apoptosis.

It is well documented that STAT3 is phosphorylated for tumor progression and inflammation by receptor-associated Janus kinases (JAK) [52, 53]. Interestingly, PROS attenuated phosphorylation of STAT3 in A549 and H460 cells and also reduced the expression of IL6 activated p-ERK and p-STAT3 in STAT3 low level H1299 cells, demonstrating the potent role of STAT3 in PROS induced apoptosis.

Accumulating evidences reveal that targeting angiogenesis is a good strategy for effective anticancer therapy [54, 55]. Also, STAT3 is closely associated with VEGF [56, 57] or CDK2 [58] in cancer progression. Here, PROS inhibited VEGF induced proliferation, migration and tube formation in HUVECs and suppressed angiogenesis in CAM assay. Furthermore, PROS attenuated the expression of p-STAT3, VEGF and CDK2 and also disrupted the binding of STAT3 with CDK2 or VEGF in A549 cells, indicating PROS exerts antiangiogenic effect via disrupted binding of STAT3 with CDK2 or VEGF. Additionally, PROS reduced the growth of H460 cells implanted in BALB/c athymic nude mice via inhibition of STAT3, VEGF and activation of caspase 3.

**Figure 6:**
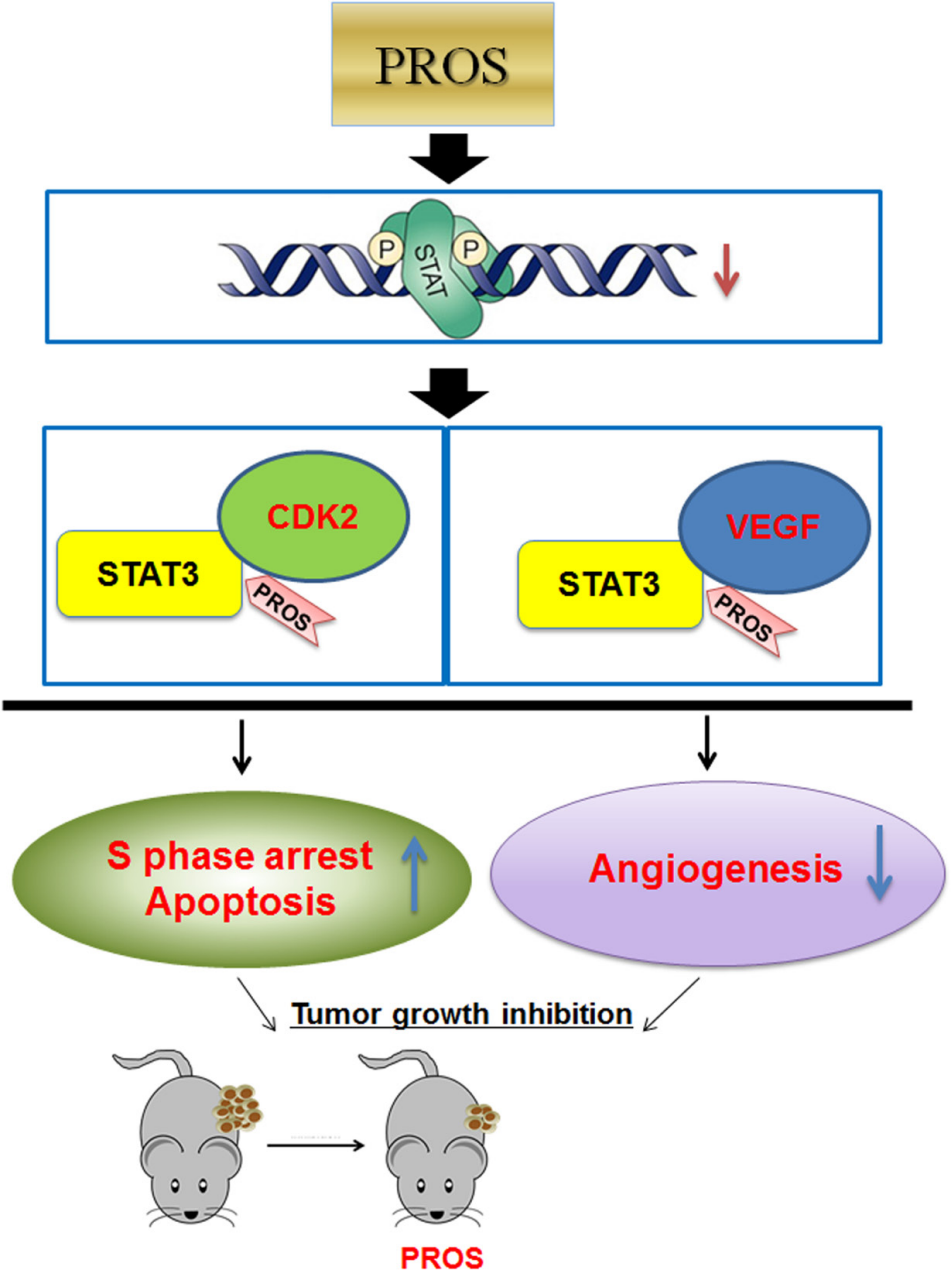
Schematic diagram of antiangiogenic and apoptotic effect of PROS via inhibition of STAT3/VEGF/CDK2 axis signaling

In summary, PROS showed significant cytotoxicity, sub G1 accumulation and S phase arrest, via inhibition of pro-PARP, Bcl-2, Cyclin E, Cyclin A, CDK2, E2F1, p-Src, p-STAT3, p-ERK, p-AKT, COX-2 and SOCS-1 along with disrupted binding of STAT3 with CDK2 or VEGF in NSCLCs. Furthermore, PROS inhibited VEGF induced proliferation, migration and tube formation in HUVECs and *ex vivo* angiogenesis in CAM via reduced phosphorylation of VEGFR2, Src and STAT3. Also, PROS reduced the growth of H460 cells in nude mice via inhibition of STAT3, and VEGF and activation of caspase 3. Overall, these findings demonstrate that PROS exerts antiangiogenic and apoptotic effects via inhibition of STAT3/VEGF/CDK2 axis signaling as a potent anticancer agent for lung cancer treatment (Figure 6).

## MATERIALS AND METHODS

### Preparation of PROS

Tetraarsenic hexoxide (As_4_O_6,_ MW = 395.6828;PR) from processed *Realgar* was provided from Chonjisan Networks. *Oldenlandia diffusa* (25 g) and *Salvia miltiorrhiza* (10g) were extracted with 50% EtOH for 3 days at room temperature, concentrated using a rotary evaporator (Eyela, Tokyo, Japan) and freeze-dried to obtain 4.64 g (yield = 13.25%) of ethanol extracts of *Oldenlandia diffusa* and *Salvia miltiorrhiza* (OS). PROS consists of 2.5 μg/ml tetraarsenic hexoxide (PR) and OS herbal mixture (180 μg/ml).

### Cell culture

Human non-small lung cancer cell lines such as A549 (ATCC^®^ ccl-185^™^) and H460 (ATCC^®^ HTB-177^™^) and H1299 (ATCC^®^ CRL-5803^™^) cells were obtained from American Type Culture Collection (ATCC) and maintained in RPMI1640 (Welgene, Daegu, Republic of Korea) supplemented with 10% FBS and penicillin/streptomycin. Human umbilical vein endothelial cells (HUVECs) were isolated from fresh human umbilical cord vein by collagenase treatment. The cells were cultured in M199 supplemented with 20% heat inactivated FBS, 3 ng/ml bFGF, 5 units/ml heparin and 100 units/ml antibiotic-antimycotic on 0.1% gelatin-coated flasks at 37°C in a humidified atmosphere containing 5% CO_2_. HUVECs were used within passages three to six.

### Cytotoxicity assay

The cytotoxicity of PR, OS and PROS was measured by 3-(4,5-dimethylthiazol-2-yl)-2,5-diphenyltetrazolium bromide (MTT) assay. In brief, A549 and H460 cells (1 × 10^4^ cells/well) were seeded onto 96-well culture plate and exposed to various concentrations of PR or/and OS (180 μg/ml) for 24 h, while HUVECs were exposed only to PROS for 24 h. The cells were incubated with MTT (1 mg/mL) (Sigma Chemical) for 2 h and then treated with MTT lysis solution overnight. Optical density (OD) was measured using a microplate reader (Molecular Devices Co., USA) at 570 nm. Cell viability was calculated as a percentage of viable cells in drug treated groups versus untreated control.

### Cell cycle analysis

A549 cells (1 × 10^6^ cells/ml) were treated with PR (2.5 μg/ml) or/and OS (180 μg/ml) for 24 h, washed twice with cold PBS and fixed in 75% ethanol at −20°C. The cells were incubated with RNase A (10 mg/ml) for 1 h at 37°C and stained with propidium iodide (50 μg/ml) for 30 min at room temperature in dark. The stained cells were analyzed for the DNA content by FACSCalibur (Becton Dickinson, Franklin Lakes, NJ, USA) using Cell Quest Software.

### DAPI staining and microscopic observation

A549 and H460 cells were treated with PR (2.5 μg/ml) or/and OS (180 μg/ml) for 24 h and the treated cells were seeded onto poly-L-lysine coated slides and fixed using 4% methanol-free formaldehyde solution for 25 min at 4°C. After washing the slides with PBS, mounting medium with DAPI was dispersed over the entire section of slides and visualized under AxioVision 4.0 fluorescence microscope (Carl Zeiss Inc., Weimar, Germany).

### VEGF induced proliferation assay

HUVECs (1 × 10^4^ cells) were seeded onto 0.1% gelatin-coated 96-well microplates and incubated in a humidified incubator for 24 h. the cells were cultured in M199 containing 5% heat-inactivated FBS, 10 ng/ml VEGF the presence or absence of PROS for 48 h and the viability of HUVECs was determined by using MTT assay.

### VEGF induced migration assay

Confluent HUVECs (3 × 10^5^ cells) were seeded onto 0.1% gelatin coated 6-well plates and the monolayer was wounded by using a tip of a 200ml pipette tip. Then cells were treated with PROS in M199 with 5% FBS, 10 ng/ml VEGF and 5 units/ml heparin for 18 h. Then the cell were rinsed with PBS and stained with Diff Quick solution and randomly chosen fields were photographed under a light microscope at X100 magnification. The number of migrated cells was counted.

### Wound healing assay

The motility of A549 cells was assayed by a wound-healing assay. Cells (4 × 10^5^ cells/mL) were seeded onto 6-well plate and incubated at 37°C. The confluent cells were scratched with a 200 μL pipet tip, followed by washing with PBS, and then treated with PR (2.5 μg/ml) or/and OS (180 μg/ml) in a complete medium for 16 h. Then the cells were fixed and stained with Diff-Quick, and randomly chosen fields were photographed under a fluorescence microscope (AXIO observer A1, Zeiss, Germany). The number of cells migrated into the scratched area was calculated.

### VEGF induced tube formation assay

*In vitro* differentiation of HUVECs into capillary-like tubes was evaluated on Matrigel. HUVECs (2 × 10^4^ cells) were seeded onto Matrigel-coated 24-well plates, and were cultured in M199 with 1% FBS, 10 ng/ml VEGF and 5 units/ml heparin with or without PROS for 18 h. Then, cells were fixed with 4% paraformaldehyde, and randomly chosen fields were photographed under an Axiovert S 100 light microscope (Carl Zeiss, Thornwood, NY, U.S.A.) at ×100 magnification.

### Chorioallantoic membrane assay

The *ex vivo* angiogenic activity was assessed using chorioallantoic membrane (CAM) assay. PROS and VEGF (100 ng) were loaded onto 1/4 piece of thermonox disk (Nunc, Naperville, IL, USA). The dried thermonox disk was applied to the CAM of a 10-day-old embryo. After 2 days incubation, a fat emulsion was injected under the CAM for better visualization of the blood vessels. The number of newly formed blood vessels was counted.

### Immunoprecipitation

A549 and H460 cells were lyzed in lysis buffer (50 mM Tris–HCl, pH 7.4, 150 mM NaCl, 1% Triton X-100, 0.1% SDS, 1 mM NaF, 1 mM EDTA, 1mM Na_3_VO_4_, and 1× protease inhibitor cocktail), and then were immuneprecipitated with STAT3, CDK2 and VEGF antibodies. Thereafter, Protein A/G sepharose beads (Santa Cruz Biotechnology, Santa Cruz, CA, USA) were applied. The final precipitated proteins were subjected to immunoblotting with the indicated antibodies.

### Western blotting

A549 and H460 cells (1 × 10^6^ cells/ml) were treated with PROS for 24 h, lyzed in lysis (50 mM Tris–HCl, pH 7.4, 150 mM NaCl, 1% Triton X-100, 0.1% SDS, 1 mM EDTA, 1 mM Na_3_VO_4_, 1 mM NaF, and 1× protease inhibitor cocktail) on ice, and spun down at 14,000×*g* for 20 min at 4°C. The supernatants were collected and quantified for protein concentration by using RC DC protein assay ki (Bio-Rad, Hercules, CA, USA). The protein samples were separated on 4–12% NuPAGE Bis–Tris gels (Novex, Carlsbad, CA, USA) and transferred to a Hybond ECL transfer membrane for detection with antibodies for PARP, cleaved-PARP, Caspase3, Bcl-2, p53, p21, cyclin E, Cyclin A, CDK2, E2F-1, p-VEGFR2, VEGFR2, phospho-Src, phospho-AKT, cyclinD1, phospho-JNK, JNK, phospho-p38 MAPK, p38 MAPK, phospho-ERK, ERK, phospho-STAT3, STAT3 and SHP-1 (Cell signaling Technology, Beverly, MA), SOCS-1, COX-2 and VEGF (Santa Cruz Biotechnologies, Santa Cruz, CA, USA), and β-actin (Sigma, St. Louis, MO, USA).

### H460 xenograft model

The animal studies were conducted under guidelines approved by Institutional Animal Care and use Committee, Kyung Hee University [KHUASP(SE)-17-003]. Briefly, 2 million H460 cells were mixed with Matrigel (50%, in 100 μl; Becton Dickinson, Bedford, MA, USA) and injected subcutaneously on the right flank of 6-week-old female BALB/c athymic nude mice (NARA Biotech, Seoul, Korea). One day after tumor inoculation, mice were daily given oral administration of PROS (PR 125 μg/kg + OS 20 mg/kg) for 18 days. Tumor volumes were measured every six days with a caliper, and calculated. According to the formula [(L × W^2^)/2], where L and W stand for length and width, respectively. All mice were sacrificed 18 days after tumor inoculation and the tumors were excised and weighed.

### Immunohistochemistry

Tumors were fixed in 10% neutral buffered formalin for 16 h, embedded in paraffin, and sectioned to 4 μm thickness. The tumor sections were immobilized and deparaffinized by immersing in xylene, dehydrated in a graded series of ethanol and washed with distilled water. For antigen retrieval, the tumor sections were boiled in 10 mM sodium citrate buffer (pH 6.0) for 10 min and cooled at room temperature. After washing with Tris-buffered saline (TBS), endogenous peroxidase activity was blocked by incubation in 3% H_2_O_2_–methanol for 10 min at room temperature. The sections were stained with antibodies for p-STAT3 (Cell Signaling Technology, Beverly, MA, USA), cleaved-caspase-3 (Cell Signaling Technology, Beverly, MA, USA) and VEGF (Santa Cruz Biotechnologies, Santa Cruz, CA, USA) over-night at 4°C using ABC and DAB kits (Vector Lab., Inc., Burlingame, CA, USA) and also counterstained with Mayer's hematoxylin solution (Sigma chemical Co., St. Louis, MO, USA). All stained sections were photographed under an Axiovert S 100 light microscope (Carl Zeiss, Inc., USA) at X200 magnifications.

### Statistical analysis

The results were expressed as the means ± SD from at least three independent experiments. Statistical analyses were conducted by Student's *t*-test using Graphpad Prism 5.0 software (GraphPad Software, San Diego, CA, USA). *P* value < 0.05 was considered statistically significant.

## Abbreviations

EGFR: Epidermal growth factor receptor
VEGF: vascular endothelial growth factor
ALK: Anaplastic lymphoma kinase
NF-κB: nuclear factor kappa-light-chain-enhancer of activated B cells
PARP: poly (ADP-ribose) polymerase
Bcl-2: B-cell lymphoma 2
ERK: extracellular signal-regulated kinase
CDK2: cyclin-dependent kinase 2
E2F-1: E2F transcription factor 1
STAT3: signal transducer and activator of transcription 3
IL-6: interleukin 6
SHP-1: Src homology region 2 domain-containing phosphatase-1
SOCS-1: suppressor of cytokine signaling 1
COX-2: cyclooxygenase-2
CAM: chorioallantoic membrane
VEGFR2: vascular endothelial growth factor receptor 2
HUVECs: human umbilical vein endothelial cells
bFGF: basic fibroblast growth factor
MTT: 3-(4,5-dimethylthiazol-2-yl)-2,5-diphenyltetrazolium bromide
PBS: phosphate buffered saline
DAPI: ,4′,6-diamidino-2-phenylindole
SDS: sodium dodecyl sulfate (SDS)
APL: acute promyelocytic leukemia.
